# Depth of implantation in relation to membranous septum as a predictor of conduction disturbances after transcatheter aortic valve implantation

**DOI:** 10.1016/j.ipej.2024.03.003

**Published:** 2024-03-26

**Authors:** Mahmoud Baraka, Diaa Kamal, Ahmad E. Mostafa

**Affiliations:** Department of Cardiology, Ain Shams University, Cairo, Egypt

**Keywords:** TAVI, Conduction disturbances, AV block, LBBB, Depth of implantation

## Abstract

**Background:**

Conduction disturbances remain one of the most common complications occurring post TAVI. We aim to determine the predictors of cardiac conduction disturbances after Transcatheter Aortic Valve Implantation (TAVI) and propose a relevant predictive model.

We included 70 consecutive patients with severe symptomatic AS who underwent TAVI using the self-expanding valve Evolut R or the balloon expandable Sapien XT valve. All patients were subjected to electrocardiographic evaluation pre- and post-TAVI and at 30 days. Clinical, echocardiographic, CT-derived, and procedural parameters were collected and analyzed.

**Results:**

Conduction disturbances affected 28 patients (40%): 16 patients (22.9 %) developed Left Bundle Branch Block (LBBB), 7 patients (10%) experienced transient Complete Heart Block (CHB), and 5 patients (7.1%) experienced permanent CHB requiring Permanent Pacemaker Implantation (PPI). We classified predictors into preprocedural and procedural predictors. Multivariate logistic regression analysis of pre-procedural predictors showed that the presence of basal septal calcification is the most powerful independent predictor (OR: 28.63, 95% CI: 4.59–178.68, p < 0.001). Multivariate logistic regression analysis for pre and post procedural predictors showed that the relationship between depth of implantation at the septum and membranous septum expressed in percentage (sDIMS) with cut-off >70.42% is the most powerful independent procedural predictor (OR: 1.11, 95% CI: 1.03–1.2, p 0.006).

**Conclusion:**

Conduction disturbances remain a common complication of TAVI. Presence of basal septal calcification is a non-modifiable risk factor that increase patient propensity of development such complication after TAVI. A depth of implantation exceeding 70% of the membranous septal length has been found to strongly predict conduction disturbances post TAVI. sDIMS can be used in planning the depth of implantation to reduce incidence of conduction disturbances post TAVI.

## 1 Background

In less than 20 years of its conception, Transcatheter Aortic Valve Implantation (TAVI) has gradually replaced Surgical Aortic Valve Replacement (SAVR) as the indicated modality of treatment for calcific aortic stenosis (AS) in old age [1,2]. Despite TAVI proving its non-inferiority in mortality and debilitating stroke compared to SAVR in patients with high or intermediate surgical risk [[3], [4], [5], [6]], allowing further extension of the indication to lower risk patients, conduction disturbances remain higher post TAVI compared to SAVR [7,8]. Identifying predictors for the occurrence of conduction disturbances is still lacking and can be the first step to decrease its incidence post TAVI.

The close interaction between aortic root complex and membranous part of the interventricular septum, which in turn is intimately related to the Atrio-Ventricular Bundle (AVB) after it exits the Atrio-Ventricular Node (AVN), can explain the increased frequency of conduction disturbances post TAVI [9]. A deeper implantation of *Trans*-catheter Heart Valve (THV) has been found to increase the risk of conduction disturbances post TAVI as it increases the risk of injury of AVB as it surfaces below the membranous septum [10,11]. Furthermore, the interaction between membranous septum and depth of implanted THV seems to play an important role in prediction of conduction disturbances post TAVI [12]. A variable anatomical interaction between the course of the AVB and the membranous septum adds another layer of complexity to this interaction [13].

The aim of our study is to determine the predictors of cardiac conduction disturbances after TAVI and propose a relevant predictive model.

## 2 Methods

### 2.1 Study design

Prospective single center observational study conducted to all comers who receive TAVI at our institute.

### 2.2 Patient selection

All comers, from a period of January 2020 to November 2021 in a single center, were evaluated for enrollment in our study. Seventy-three patients presented for TAVI at our institute, however 2 of which had LBBB and one had a permanent pacemaker already implanted. The study therefore included 70 consecutive patients with severe symptomatic AS; defined as AVA <1 cm^2^ or <0.6 cm^2^/m^2^, with or without aortic regurgitation and have aortic valve annulus diameter ≥18 and ≤ 29 mm. Patients with previous pacemaker insertion, pre-existing LBBB, estimated life expectancy <1-year, active endocarditis, LV thrombus were excluded.

Patients underwent clinical evaluation, baseline ECG, and contrast CT prior to TAVI, followed by follow-up ECG and clinical status. All parameters recorded were divided into pre- and post-procedural factors.

### 2.3 ECG data

A standard 12 lead ECG was obtained upon admission to the hospital prior to the procedure, with PR interval, and QRS duration calculated, as well as identifying RBBB, LBBB or IVCD. Electrocardiographic outcomes were assessed continuously during the procedure. After the procedure, the patients underwent continuous monitoring of heart rhythm for average of 2 days. After 1 month of the procedure, another standard 12 lead surface ECG was recorded to determine the final electrical outcomes post TAVI.

### 2.4 Echocardiographic data

Baseline data on LV dimensions, ejection fraction and thickness of septal and posterior walls of LV, as well as measurement of peak and pressure gradient and calculate Aortic Valve Area (AVA) using continuity equation. All patients underwent their echocardiographic studies using GE Vivid 7 machines with online analysis during the study.

### 2.5 CT data

All patients underwent contrast MSCT (Multi-Slice Computed Tomography) study, which was done using a dual source CT machine (Somatom Definition Flash, Siemens Healthineers, Forchheim, Germany) using two x-ray tubes and 2 detectors arranged at an angular offset of 95°. Each detector enables data acquisition with 64 rows of detectors of 0.6 mm width (Z-axis coverage: 38.4 mm). Together with a Z-flying focal spot allowing simultaneous acquisition of data in 2 x 128 slices. With a gantry rotation time of 0.28 s, half-scan reconstruction yields a temporal resolution of 75 ms in the center of the field of view. Patients with heart rate ≥70 bpm received a beta-blocker (Atenolol 50–100 mg) orally half an hour before the study to control heart rate to be 60–70 bpm. Estimation of individual circulation time was based on the test bolus technique using a 10 ml bolus contrast.

Contrast enhancement was achieved through automated injection of 60–80 ml iopromide (370 mg I/ml Ultravist®, Bayer Schering Pharma AG, Germany) by a power injector at a flow rate of 5 ml/s plus a 40 ml saline flush. Imaging was done using 120 kV tube voltage and tube current was set according to body mass index (BMI). All CT imaging data were acquired in deep inspiration.

Datasets were acquired using prospectively ECG-triggered high-pitch spiral acquisition (Flash technology) for all patients triggered at 60% of the R–R interval in a craniocaudal direction scanning the area from the root of the neck down to 5 cm below femoral heads. Datasets were reconstructed at a slice thickness of 0.6 mm to assess cardiac structures and 1 mm to assess the entire vascular access. ECG was digitized and continuously monitored during the scanning period.

Acquired datasets were transmitted and analyzed using dedicated software (Syngo.via®, Siemens Healthineers or OsiriX MD® DICOM Viewer) to get multiplanar reconstructions (MPRs). The standard sagittal and coronal views were used for initial orientation at the level of the aortic valve to generate double-oblique axial images to get the proper plane connecting the nadir of the 3 aortic cusps representing the plane of the hemodynamic annulus. MS length was measured as the maximum seen part from the commissure between non-coronary and right coronary cusps cranially to the basal muscular IVS caudally in the reformatted coronal view. Standard recorded CT parameters included annular measurements: minimum and maximum diameters, perimeter, and area with the respectively derived diameters; as well as height of coronary arteries from the annular plane, width of sinuses of Valsalva and distribution of calcification in the aortic root complex. The CT analysis was done independently by two members of the clinical team, trained in cardiac CT analysis with no significant interobserver variability.

### 2.6 TAVI procedure

TAVI was done using the self-expandable valve Evolut R or the balloon expandable Sapien XT valve through femoral access using their corresponding sheaths and delivery systems. The procedure was performed with local anesthesia in combination with a mild systemic sedative/analgesic treatment. Vascular access was obtained percutaneously through the common femoral artery (with or without pre-planned surgical cutdown according to availability of vascular closure devices at our center). At the start of each procedure, a temporary transvenous pacemaker was positioned in the right ventricle through transjugular or transfemoral access. This pacemaker remained in position for at least 24 h after TAVI and was removed when there were no signs of AV block or bradycardia. Type and size of used THV were recorded as well as the need for pre-dilatation or post-dilatation.

To measure the depth of implantation at the septal side (sDI), a final cine loop is obtained in the pre-determined angle of implantation, with occasional slight cranial or caudal angulations to remove the parallax, using 25 mL of contrast to outline the nadirs of the 3 aortic cusps and help measure the depth of implantation from a reference level. Below this level and into Left Ventricular Outflow Tract (LVOT) side, the extension of THV frame is measured in millimeters, after calibration, using the respective software at the catheter lab (Philips and Siemens) as shown in Fig. I.

**Fig. 1:**
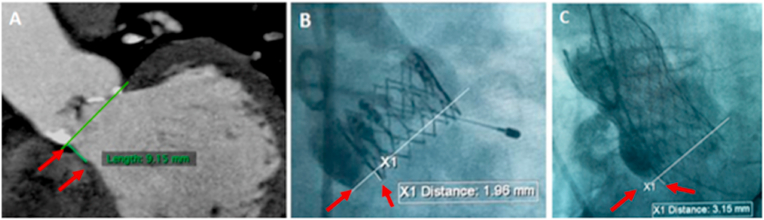
(A) CT coronal view showing length of membranous septum length measured as a straight line joining the nadir of NCC at the virtual annulus of the AV and the crest of the muscular septum**, (B and C)** Fluoroscopy views showing the depth of implantation of Sapien XT and Evolut R valves respectively measured as a straight line from the lower most point of the NCC and the lower edge of the implanted valve.

### 2.7 Relationship between length of membranous septum and depth of implantation of the THV

The length of membranous septum as measured by MSCT, as well as depth of implantation of THV from septal side measured by fluoroscopy during the procedure, are both used to derive two parameters that explain the relationship of length of the membranous septum and the depth of implantation from septal side (Fig. II). The first is derived by arithmetic subtraction of the sDI from MS, which would be called ΔMSID. The second parameter is a percentage derived by dividing the sDI by MS; this parameter was thought of in order to overcome the anatomical variations in the length of the membranous septum among patients, which we called sDIMS (sDI/MS x 100).

**Fig. 2:**
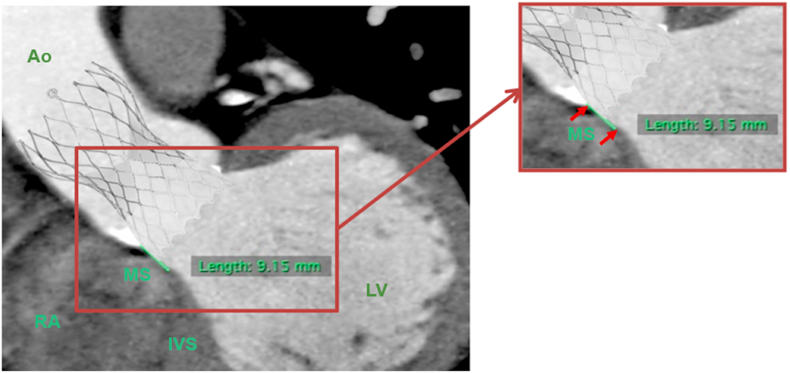
Illustration showing the relationship between the depth of implantation and the membranous septum; Pre-TAVI CT showing membranous septum length equals to 9.15 mm (represented by the green line) and a graphical overlay of an Evolut valve imitating the depth of implantation. (Ao: aorta, RA: right atrium, MS: membranous septum, IVS: muscular part of interventricular septum, LV: left ventricle).

### 2.8 Statistical analysis

The collected data was revised, coded, tabulated, and introduced to a PC using Statistical package for Social Science (SPSS 25). Parametric numerical data is described using mean, standard deviation (SD) and range, and analyzed using student *t*-test. Median and interquartile range (IQR) were used for description of non-parametric numerical data, and analysis was done using Mann-Whitney test. Non-numerical data are represented by frequency and percentage. The Kruskal-Wallis test was used to assess the statistical significance of the difference between more than two study group ordinal variables and Chi-square test was used to examine the relationship between two qualitative variables. Fisher's exact test was used to examine the relationship between two qualitative variables when the expected count is less than 5 in more than 20% of cells. The Receiver Operating Characteristics (ROC) curve was used to provide sensitivity and specificity for predictors that were found significant in the univariate analysis. Results are presented as hazard ratio with 95% confidence interval and a p < 0.05 was considered statistically significant. Parameters that were found significant in the univariate analysis were used to create a multi-variate regression model.

## 3 Results

### 3.1 Pre-procedural parameters and patients’ characteristics

Conduction disturbances were seen in 28 patients (40%); 16 patients (22.9%) experienced LBBB, 7 patients (10%) experienced transient high-grade AV block with spontaneous recovery <48 h post-TAVI, and 5 patients (7.1%) experienced permanent CHB requiring permanent pacemaker implantation (PPI). Summary of the patients’ characteristics, clinical data, and pre-procedural ECG, echo and CT parameters with their univariate analysis is seen in Table 1, Table 2, Table 3. The single pre-procedural predictor that showed significance on univariate analysis was the presence of basal septal calcification (p < 0.001).

**Table 1:** Distribution of results and univariate analysis of demographic and clinical parameters.

Parameters	Conduction disturbance Absent (N = 42	Conduction disturbance Present (N = 28	P Value
Age (years)	75.71 ± 6.88	74.36 ± 7.53	†0.439
Male sex	23 (54.76%)	16 (57.14%)	φ 0.844
Body mass index (kg/m^2^)	29.58 ± 5.64	29.65 ± 8.19	†0.964
Body surface area (m^2^)	1.89 ± 0.2	1.9 ± 0.25	†0.951
EuroSCORE II (%)	5.88 (2.87–8.56)	6.54 (3.56–11.23)	†0.24
Smoking	12 (28.57%)	4 (14.29%)	φ 0.163
Creatinine clearance (ml/min)	56 (39–79)	48.5 (40.5–83)	‡ 0.895
Diabetes Mellitus	21 (50%)	12 (46.86%)	φ 0.558
Hypertension	32 (76.19%)	20 (71.43%)	φ 0.655
Ischemic heart disease	21 (50%)	14 (50%)	φ 1
Previous cerebrovascular stroke	9 (21.43%)	7 (25%)	φ 0.727
CABG	6 (14.29%)	1 (3.57%)	φ 0.23
Chronic lung disease	8 (19.05%)	6 (21.43%)	φ 0.807

†Independent *t*-test, φ Chi square test, φ Fisher's Exact test; ‡ Mann-Whitney test, CABG: coronary artery bypass graft.

**Table 2:** Distribution of results and univariate analysis of ECG and echocardiographic parameters.

Parameters	Conduction disturbance Absent (N = 42)	Conduction disturbance Present (N = 28)	p value
ECG parameters			
Atrial fibrillation (AF)	3 (7.14%)	6 (21.43%)	φ 0.142
RBBB	2 (4.76%)	6 (21.43%)	φ 0.052
PR interval duration (msec)	180 ± 37.13	179.09 ± 34.49	† 0.925
QRS duration (msec)	94.76 ± 18.77	105 ± 24.87	† 0.054
Echocardiographic Parameters			
Ejection Fraction (%)	61.36 ± 12.87	56.43 ± 16.55	† 0.166
SWT (mm)	13.02 ± 1.99	13.3 ± 2.01	† 0.569
SWT - indexed (mm/m^2^)	6.94 ± 1.23	7.08 ± 1.1	† 0.63
PWT (mm)	12.45 ± 1.52	12.58 ± 1.78	† 0.763
PWT - indexed (mm/m^2^)	6.64 ± 1.05	6.7 ± 1.04	† 0.816
LVEDD (mm)	51.38 ± 5.87	51.36 ± 8.14	† 0.989
LVEDD - indexed (mm/m^2^)	26.75 ± 6.19	27.37 ± 4.76	† 0.661
LVESD (mm)	33.07 ± 7.74	34.46 ± 9.59	† 0.505
LVESD - indexed (mm/m^2^)	17.63 ± 5.18	18.41 ± 5.5	† 0.549
Mean pressure gradient (mmHg)	50.29 ± 12.47	47.96 ± 8.24	† 0.354
AVA (cm^2^)	0.74 ± 0.17	0.75 ± 0.18	† 0.813
AVA - indexed (cm^2^/m^2^)	0.4 ± 0.09	0.4 ± 0.1	† 0.992

†Independent *t*-test, φ Fisher's Exact test, SWT: basal interventricular septal wall thickness; PWT: basal posterior wall thickness; LVEDD: left ventricular end-diastolic diameter, LVESD: left ventricular end systolic diameter; AVA: Aortic Valve Area.

**Table 3:** Distribution of results and univariate analysis of CT-derived parameters.

Findings	Conduction disturbance Absent (N = 42)	Conduction disturbance Present (N = 28)	p value
Annulus mean diameter (mm)	23.96 ± 1.79	23.36 ± 2.64	† 0.294
Annulus mean diameter - indexed (mm/m^2^)	12.75 ± 1.3	12.46 ± 1.71	† 0.427
Annulus perimeter (mm)	76.65 ± 5.46	74.17 ± 8.08	† 0.129
Annulus perimeter indexed (mm/m^2^)	40.77 ± 4.2	39.58 ± 5.49	† 0.306
Annulus area (mm^2^)	444.57 ± 63.22	419.04 ± 86.54	† 0.158
Annulus area indexed (mm^2^/m^2^)	235.52 ± 33.75	222.56 ± 43.94	† 0.168
LMCA height (mm)	12.85 ± 2.12	12.69 ± 1.58	† 0.721
LMCA indexed height (mm/m^2^)	6.83 ± 1.2	6.8 ± 1.24	† 0.921
RCA height (mm)	13.69 ± 2.95	13.31 ± 2.1	† 0.567
RCA indexed height (mm/m^2^)	7.29 ± 1.69	7.12 ± 1.33	† 0.648
MS (mm)	7.94 ± 2.21	7.48 ± 2.03	† 0.376
MS - indexed (mm/m^2^)	4.24 ± 1.29	3.97 ± 1.08	† 0.377
Basal septal calcification	2 (4.76%)	14 (50%)	φ < 0.001*

†Independent *t*-test, φ Chi square test, *significant.

LMCA: left main coronary artery; RCA: right coronary artery; MS: length of membranous septum.

### 3.2 Procedural parameters

Table IV illustrates the univariate analysis of different procedural parameters and their correlation with conduction disturbances post TAVI showing the significance of depth of implantation of the THV on the results, alone or when compared to length of membranous septum measures pre-procedurally (see Table 5).

**Table 4:** Distribution of results and univariate analysis of procedural parameters.

Characteristics		Conduction disturbance Absent (N = 42	Conduction disturbance Present (N = 28	p
**Valve Type**	Evolut R	39 (95.1%)	21 (72.4%)	
	Sapien XT	2 (4.87%)	8 (27.5%)	
	Self-expanding valves	33 (78.57%)	26 (92.86%)	φ 0.18
	Balloon-expandable valves	9 (21.43%)	2 (7.14%)	
**Pre-dilatation**		14 (33.33%)	4 (14.29%)	φ 0.074
**Post-dilatation**		4 (9.52%)	7 (25%)	φ 0.102
**sDI (mm)**		3.39 ± 1.5	6.58 ± 3	† <0.001*
**sDI - indexed (mm/m**^**2**^**)**		1.81 ± 0.78	3.49 ± 1.57	† <0.001*
**sDIMS (%)**		44.79 ± 17.95	88.55 ± 30.61	† <0.001*
**ΔMSID (mm)**		4.11 (2.57–5.7)	1.08 (0.05–2.44)	‡ <0.001*

†Independent *t*-test, φ Chi square test, φ Fisher's Exact test, ‡ Mann-Whitney test *significant sDI: depth of implantation of THV measured at the septal side; sDIMS: ratio between sDI and length of membranous septum; ΔMSID: difference between membranous septum length and sDI.

**Table 5:** Diagnostic performance of the significant numeric parameters in predicting conduction abnormalities.

Factors	AUC	P	95% CI	Cut off
sDI	0.844	<0.001*	0.737 to 0.919	>4.85 mm
sDI - indexed	0.836	<0.001*	0.728 to 0.914	>2.12 mm/m^2^
sDIMS	0.903	<0.001*	0.808 to 0.961	>70.42%
ΔMSID	0.832	<0.001*	0.724 to 0.911	≤2.38 mm

AUC: Area under curve, SE: Standard error, CI: Confidence interval, *significant sDI: depth of implantation of THV, measured at the septal side; sDIMS: ratio between sDI and length of membranous septum; ΔMSID: difference between membranous septum length and sDI.

### 3.3 Multivariate regression model

We performed two multivariate regression models, a pre-procedural model consisting of basal septal calcification which showed significant correlation with p < 0.001, and we also included two other ECG parameters which were approaching significance; pre-existing RBBB and QRS duration with p value 0.052 and 0.054 respectively. This model showed that basal septal calcification is the most powerful independent predictor (OR: 28.63, 95% CI: 4.59–178.68, p < 0.001) as shown in Table VI.

**Table 6:** Multivariate logistic regression analysis for pre-procedural parameters.

Pre-procedural Parameters	β	SE	P	OR (95% CI)
Basal septal calcification	3.355	0.934	<0.001*	28.63 (4.59–178.68)
RBBB	−1.676	1.783	0.347	0.19 (0.01–6.17)
QRS duration	0.043	0.025	0.093	1.04 (0.99–1.1)

β: Regression coefficient; SE: Standard error; OR: Odds ratio; CI: Confidence interval; *significant; RBBB: right bundle branch block.

The other model included pre and post procedural parameters and concluded that sDIMS >70.42% is the most significant independent factor predicting conduction disturbances post TAVI (OR: 1.11, 95% CI: 1.03–1.2, p 0.006) as shown in Table VII.

**Table 7:** Multivariate logistic regression analysis for pre and post procedural parameters.

Pre and post procedural parameters	β	SE	P	OR (95% CI)
Basal septal calcification	2.129	1.473	0.148	8.41 (0.47–150.91)
RBBB	−3.867	2.393	0.106	0.02 (0–2.28)
QRS duration	0.084	0.042	0.064	1.09 (0.98–1.17)
sDIMS >70.42%	0.107	0.039	0.006*	1.11 (1.03–1.2)
ΔMSID <2.38 mm	0.288	0.299	0.336	1.33 (0.74–2.4)

β: Regression coefficient; SE: Standard error; OR: Odds ratio; CI: Confidence interval; *significant; RBBB: right bundle branch block; DIMS: percentage of DI of membranous septum; ΔMSID: difference between membranous septum length and sDI.

## 4 Discussion

The principal findings of this study are [1]: Basal septal calcification, detected by CT, in the vicinity of the membranous septum, is the most significant pre-procedural predictor of conduction disturbances post TAVI [2]. Depth of implantation of THV is a significant factor in occurrence of post TAVI conduction disturbances. A percentage ratio of depth of implantation as measured from the NCC in CT to the total length of the MS >70.42% has shown to be the most significant predictor of conduction disturbances in a model that includes all pre- and post-procedural factors.

Whilst efforts to reduce the occurrence of complications after TAVI have resulted in improvements in valves technology with a substantial reduction of their severity and their clinical impact [[14], [15], [16]], the development of conduction disturbances after TAVI has failed to decrease significantly in recent times despite the development of newer-generation valves [4,7,8,[17], [18], [19], [20]]. Incidence of conduction disturbances in our study match the international rates, especially for self-expanding platforms [8,21].

As regards the procedural –modifiable- risk factors and depth of implantation, one study (n = 65; CoreValve only) reported a frame depth in the LVOT of 11.1 mm as an independent predictor of PPI with 81% sensitivity and 84.6% specificity [22]. Similarly, another study revealed that if the proximal end of the valve frame was positioned <6.7 mm from the lower edge of the noncoronary cusp, no prosthesis-related left bundle branch block would occur [23].

The new repositionable Evolut R offers potential benefits compared to the preceding CoreValve. A study by Giannini, C. et al. comparing the performance of the Evolut R with the CoreValve showed that the recapture and reposition maneuvers allowed a less implantation depth for the Evolut R, and consequently the rate of PPI was lower in patients receiving the Evolut R. This is reflected in Medtronic recommendations of optimal DI between 4 and 6 mm for the CoreValve and later 3–5 mm for the Evolut R [24].

As regard the Sapien prosthesis, Urena, M. et al. [25] demonstrated that new-onset LBBB correlated with depth of implantation and each 1-mm increase in the depth of implantation corresponded to a 1.37 increase in the odds ratio for developing new LBBB. Although our univariate analysis has found that depth of implantation and its indexed value are significant predictors of conduction disturbances, however they were of less predictive value as parameters involving both length of membranous septum and the depth of implant in the LVOT. This can be attributed to the fact that measuring the depth of implantation as an independent factor, ignores the anatomic variation among patients, regarding the length of membranous septum and therefore the interplay between THV placed in LVOT and the cardiac conductive system.

Studying the relationship between depth of implantation (DI) and membranous septum length (MS) proved to be the most important post-procedural predictors in our study. This relationship was previously studied and expressed as the numerical difference between them (ΔMSID) in a study (N = 73) by Hamdan, A. et al. [12] using self-expanding valves, ΔMSID was shown to be the strongest independent procedural predictor of high degree AV block (OR: 1.4, 95% CI: 1.2 to 1.7, p < 0.001). Furthermore, he reached a cut–off for ΔMSID of 0.4 mm to be able to predict high degree AV block with sensitivity 92.3%, *specificity 76.7%* and negative predictive value (NPV) close to 97.8%.

Our study has shown that ΔMSID is a significant predictor of conduction disturbances, in univariate analysis, after TAVI (p < 0.001) and we reached a cut-off of ≤2.38 mm to be a strong procedural predictor of conduction disturbances with sensitivity reaching 75%, specificity 85.71%, and NPV 83.7%. The difference between cut-offs may be attributed to different study populations, as our cohort was on average younger in age and had longer membranous septum. Another point, is that depth of implantation in Hamdan et al., was calculated as the average depth measured angiographically from all 3 cusps. Meanwhile, in our study, we opted for measuring the depth of implantation only from the NCC as the most relevant in studying the interaction between patient's conductive system and depth of implantation.

Moreover, we expressed the relationship between sDI and MS in the form of percentage (sDIMS) which also turned out to be a strong predictor (p < 0.001), with a cut-off ≥70.42%. Multivariate regression analysis for pre- and post-procedural predictors, revealed sDIMS to be the most powerful independent procedural predictor (OR: 1.11, 95% CI: 1.03–1.2, p 0.006). We are proposing that expressing this relationship in the form of percentage rather than numerical difference in millimeters might be more practical and easier for use especially in situations in which the membranous septum length is short, at that point estimating and foreword planning the DI in percentage will be more convenient and feasible. In addition to that, we propose that an expression in percentage will account for possible anatomical variations in the length of membranous septum as well as the variable position of the AV bundle at which it perforates the membranous septum to cross into LV [13].

As regards pre-procedural predictors, our study concluded that basal septal calcification is the sole predictor of conduction disturbances (OR: 28.63, 95% CI: 4.59–178.68, p < 0.001). These results are in concordance with Hamdan, A. et al. [12] who also concluded that basal septal calcification is a strong independent predictor (OR: 4.9, 95% CI: 1.2 to 20.5, p = 0.031). Similarly, a study (N = 81; CoreValve) by Latsios, G. et al. [26] has found basal septal calcification as the most powerful independent predictor of conduction disturbances (OR: 1.06, 95% CI: 1.02–1.11, p 0.004). This could be attributed to the fact that calcification in the region of the aortic root can extend to the anatomically close AV bundle and its branches, causing a degenerative pathology, that can predispose to conduction disturbances on its own. Septal calcification could result in direct injury to the conduction system when it is sandwiched between the valve frame and the septum.

## 5 Conclusions

Conduction disturbances remain a common complication of TAVI. Presence of basal septal calcification is a non-modifiable risk factor that increase patient propensity of development such complication after TAVI. A depth of implantation exceeding 70% of the membranous septal length has been found to strongly predict conduction disturbances post TAVI. sDIMS can be used in planning the depth of implantation to reduce incidence of conduction disturbances post TAVI.

## 6 Limitations

This study was a single-center observational non-randomized study with all its inherent limitations. New generation balloon expandable valves were rarely used due to limited availability in Egypt at the time of our study. Post-procedural CT studies weren't done, for fear of non-indicated exposure to contrast and radiation, which limits the accuracy of measurement of depth of implantation of the THV frame into LVOT.

## Ethics committee approval and informed consent

The Ethics committee of the cardiology department of Ain Shams University has approved this research on January 8, 2020.

Informed written consent was obtained from all patients before including them in the study.

## Ethical approval and consent to participate

Approval of the Ain shams university ethical committee was obtained as it informs to the ethical guidelines of the 1964 declaration of Helsiniki as revised in 2008.

All patients were informed about the study and written consent was taken for participation.

## Consent for publication

Not applicable.

## Availability of data and materials

Data supporting the study results are available.

## Competing interest

All authors have no conflict of interest.

## Funding

This research did not receive any specific grant from funding agencies in the public, commercial, or not-for-profit sectors.

## Author contribution

Mahmoud Baraka: Conception and design of Study, Literature review, Acquisition of data, Analysis and interpretation of data, Research investigation and analysis, Data collection, Drafting of manuscript, Revising and editing the manuscript critically for important intellectual contents, Data preparation and presentation, Research coordination and management, Funding for the research, Diaa Kamal: Conception and design of Study, Literature review, Acquisition of data, Research investigation and analysis, Data collection, Drafting of manuscript, Data preparation and presentation, Supervision of the research, Research coordination and management, Funding for the research, Ahmad E. Mostafa: Conception and design of Study, Literature review, Acquisition of data, Analysis and interpretation of data, Research investigation and analysis, Data collection, Revising and editing the manuscript critically for important intellectual contents, Data preparation and presentation, Supervision of the research, Research coordination and management, Funding for the research.

## Declaration of competing interest

The authors declare that they have no known competing financial interests or personal relationships that could have appeared to influence the work reported in this paper.

## List of abbreviations

AS: Aortic stenosis
AVA: Aortic Valve Area
AVB: Atrioventricular block
AVN: Atrioventricular node
CHB: Complete heart block
IQR: Inter-Quartile Range
IVCD: Intraventricular conduction delay
IVS: Inter-Ventricular Septum
LBBB: Left bundle branch block
LCC: Left Coronary Cusp
LVOT: Left ventricular outflow tract
MS: Membranous septum
NCC: Non-Coronary Cusp
PPI: Permanent pacemaker implantation
RBBB: Right bundle branch block
RCC: Right Coronary Cusp
ROC: Receiver Operating Characteristics
SAVR: Surgical aortic valve replacement
SD: Standard Deviation
sDI: Depth of implantation at the septum
sDIMS: Depth of implantation at the septum/membranous septum x100 (percentage)
TAVI: Transcatheter aortic valve implantation
THV: Transcatheter heart valve
ΔMSID: Difference between membranous septum and depth of implantation (subtraction)

## References

[1] Otto C.M., Nishimura R.A., Bonow R.O., Carabello B.A., Erwin J.P., Gentile F. (2020). ACC/AHA guideline for the management of patients with valvular heart disease: executive summary: a report of the American college of cardiology/American heart association joint committee on clinical practice guidelines. Circulation.

[2] Vahanian A., Beyersdorf F., Praz F., Milojevic M., Baldus S., Bauersachs J. (2022). 2021 ESC/EACTS Guidelines for the management of valvular heart disease. Eur Heart J.

[3] Leon M.B., Smith C.R., Mack M., Miller D.C., Moses J.W., Svensson L.G. (2010). Transcatheter aortic-valve implantation for aortic stenosis in patients who cannot undergo surgery. N Engl J Med.

[4] Barker C.M., Reardon M.J. (2014). The CoreValve US pivotal trial. Semin Thorac Cardiovasc Surg.

[5] Leon M.B., Smith C.R., Mack M.J., Makkar R.R., Svensson L.G., Kodali S.K. (2016). Transcatheter or surgical aortic-valve replacement in intermediate-risk patients. N Engl J Med.

[6] Reardon M.J., Van Mieghem N.M., Popma J.J., Kleiman N.S., Søndergaard L., Mumtaz M. (2017). Surgical or transcatheter aortic-valve replacement in intermediate-risk patients. N Engl J Med.

[7] Mack M.J., Leon M.B., Thourani V.H., Makkar R., Kodali S.K., Russo M. (2019). Transcatheter aortic-valve replacement with a balloon-expandable valve in low-risk patients. N Engl J Med.

[8] Popma J.J., Deeb G.M., Yakubov S.J., Mumtaz M., Gada H., O'Hair D. (2019). Transcatheter aortic-valve replacement with a self-expanding valve in low-risk patients. N Engl J Med.

[9] Piazza N., de Jaegere P., Schultz C., Becker A.E., Serruys P.W., Anderson R.H. (2008). Anatomy of the aortic valvar complex and its implications for transcatheter implantation of the aortic valve. Circulation Cardiovascular interventions.

[10] Randhawa A., Gupta T., Singh P., Aggarwal A., Sahni D. (2019). Description of the aortic root anatomy in relation to transcatheter aortic valve implantation. Cardiovasc Pathol.

[11] Marzahn C., Koban C., Seifert M., Isotani A., Neuß M., Hölschermann F. (2018). Conduction recovery and avoidance of permanent pacing after transcatheter aortic valve implantation. J Cardiol.

[12] Hamdan A., Guetta V., Klempfner R., Konen E., Raanani E., Glikson M. (2015). Inverse relationship between membranous septal length and the risk of atrioventricular block in patients undergoing transcatheter aortic valve implantation. JACC Cardiovasc Interv.

[13] Kawashima T., Sato F. (2014). Visualizing anatomical evidences on atrioventricular conduction system for TAVI. Int J Cardiol.

[14] Reinohl J., Kaier K., Reinecke H., Schmoor C., Frankenstein L., Vach W. (2015). Effect of availability of transcatheter aortic-valve replacement on clinical practice. N Engl J Med.

[15] Vahl T.P., Kodali S.K., Leon M.B. (2016). Transcatheter aortic valve replacement 2016: a modern-day "through the looking-glass" adventure. J Am Coll Cardiol.

[16] Babaliaros V., Devireddy C., Lerakis S., Leonardi R., Iturra S.A., Mavromatis K. (2014). Comparison of transfemoral transcatheter aortic valve replacement performed in the catheterization laboratory (minimalist approach) versus hybrid operating room (standard approach): outcomes and cost analysis. JACC Cardiovasc Interv.

[17] Husser O., Pellegrini C., Kessler T., Burgdorf C., Thaller H., Mayr N.P. (2016). Predictors of permanent pacemaker implantations and new-onset conduction abnormalities with the SAPIEN 3 balloon-expandable transcatheter heart valve. JACC Cardiovasc Interv.

[18] De Torres-Alba F., Kaleschke G., Diller G.P., Vormbrock J., Orwat S., Radke R. (2016). Changes in the pacemaker rate after transition from edwards SAPIEN XT to SAPIEN 3 transcatheter aortic valve implantation: the critical role of valve implantation height. JACC Cardiovasc Interv.

[19] Thyregod H.G., Steinbrüchel D.A., Ihlemann N., Nissen H., Kjeldsen B.J., Petursson P. (2015). Transcatheter versus surgical aortic valve replacement in patients with severe aortic valve stenosis: 1-year results from the all-comers NOTION randomized clinical trial. J Am Coll Cardiol.

[20] Carroll J.D., Mack M.J., Vemulapalli S., Herrmann H.C., Gleason T.G., Hanzel G. (2020). STS-ACC TVT registry of transcatheter aortic valve replacement. J Am Coll Cardiol.

[21] Auffret V., Puri R., Urena M., Chamandi C., Rodriguez-Gabella T., Philippon F. (2017). Conduction disturbances after transcatheter aortic valve replacement: current status and future perspectives. Circulation.

[22] Almería Valera C., de Agustín Loeches A., Hernández-Antolín R.A., García Fernández E., Pérez de Isla L., Fernández Pérez C. (2011). Atrioventricular conduction disturbances after CoreValve ® aortic prosthesis implantation. Predictive role of transesophageal echocardiography. Rev Esp Cardiol.

[23] Piazza N., Onuma Y., Jesserun E., Kint P.P., Maugenest A.M., Anderson R.H. (2008). Early and persistent intraventricular conduction abnormalities and requirements for pacemaking after percutaneous replacement of the aortic valve. JACC Cardiovasc Interv.

[24] Giannini C., De Carlo M., Tamburino C., Ettori F., Latib A.M., Bedogni F. (2017). Transcathether aortic valve implantation with the new repositionable self-expandable Evolut R versus CoreValve system: a case-matched comparison. Int J Cardiol.

[25] Urena M., Mok M., Serra V., Dumont E., Nombela-Franco L., DeLarochelliere R. (2012). Predictive factors and long-term clinical consequences of persistent left bundle branch block following transcatheter aortic valve implantation with a balloon-expandable valve. J Am Coll Cardiol.

[26] Latsios G., Gerckens U., Buellesfeld L., Mueller R., John D., Yuecel S. (2010). "Device landing zone" calcification, assessed by MSCT, as a predictive factor for pacemaker implantation after TAVI. Cathet Cardiovasc Interv : Offic J Soc Cardiac Angiography & Interventions.

